# Supplementary material for: OSov: An Interactive Web Server to Evaluate Prognostic Biomarkers for Ovarian Cancer
Source: Biology (Basel). 2021 Dec 24;11(1):23. doi: 10.3390/biology11010023 (PMC8773055; doi:10.3390/biology11010023)
Supplement: Supplementary file 1 [file biology-11-00023-s001.zip › biology-1478768 sup final/Figure S1 OSov predicts overall survival for PLG gene for ovarian patients .pdf]

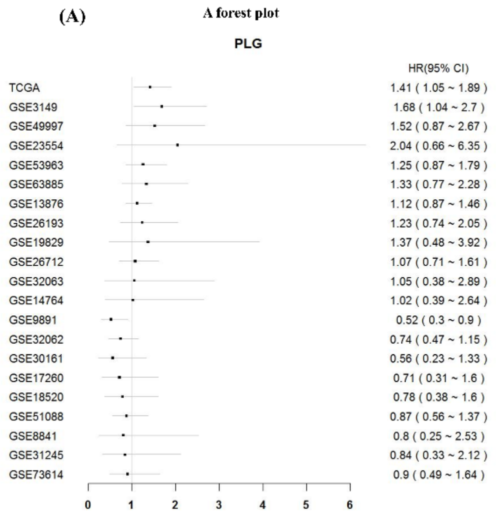

(B) Univariate and multivariate analysis

Dataset	Variables	Univariate analysis p value	Univariate HR	Univariate 95% CI	Multivariate analysis p value	Multivariate HR	Multivariate 95% CI	Outcome Uni/Multi-variable	Nomogram availably	
GSE13876	PLG	0.3733	1.12	0.87~1.46	-	-	-	-/-		Go
GSE14764	PLG	0.9722	1.02	0.39~2.64	-	-	-	-/-		Go
GSE17260	PLG	0.4075	0.71	0.31~1.60	-	-	-	-/-		Go
GSE18520	PLG	0.5050	0.78	0.38~1.60	-	-	-	-/-		Go
GSE19829	PLG	0.5515	1.37	0.48~3.92	-	-	-	-/-		Go
GSE23554	PLG	0.2165	2.04	0.66~6.35	-	-	-	-/-		Go
GSE26712	PLG	0.7483	1.07	0.71~1.61	-	-	-	-/-		Go
GSE31245	PLG	0.7130	0.84	0.33~2.12	-	-	-	-/-		Go
GSE3149	PLG	0.0323	1.68	1.04~2.70	-	-	-	Poor/-		Go
GSE26193	PLG	0.4246	1.23	0.74~2.05	0.1206	1.27	0.94~1.73	-/-	Yes	Go
	Stage	<0.0001	2.06	1.55~2.74	<0.0001	2.10	1.78~2.48	Poor/Poor		
GSE30161	PLG	0.1858	0.56	0.23~1.33	0.0695	0.64	0.40~1.04	-/-	Yes	Go
	Race	0.0989	2.44	0.85~7.06	0.0059	2.37	1.28~4.39	-/Poor		
GSE32062	PLG	0.1785	0.74	0.47~1.15	0.7181	0.97	0.80~1.16	-/-	Yes	Go
	Stage	0.0008	1.43	1.16~1.76	<0.0001	1.48	1.24~1.77	Poor/Poor		
GSE32063	PLG	0.9265	1.05	0.38~2.89	0.9189	1.02	0.65~1.60	-/-	Yes	Go
	Grade	0.1940	1.78	0.75~4.24	0.0001	2.24	1.49~3.37	-/Poor		
	Stage	0.1240	2.15	0.81~5.72	<0.0001	2.83	1.78~4.49	-/Poor		
GSE49997	PLG	0.1403	1.52	0.87~2.67	0.2035	1.45	0.82~2.56	-/-	Yes	Go
	Age	0.0172	1.89	1.12~3.19	0.0168	1.91	1.12~3.26	Poor/Poor		
	Grade	0.0324	0.46	0.22~0.94	0.0660	0.51	0.25~1.05	Good/-		
	Stage	0.0280	1.90	1.07~3.36	0.2041	1.46	0.81~2.62	Poor/-		
GSE51088	PLG	0.5581	0.87	0.56~1.37	0.7973	0.94	0.59~1.49	-/-	Yes	Go
	Age	0.1760	1.16	0.94~1.44	0.0003	2.05	1.38~3.03	-/Poor		
	Histology	<0.0001	2.21	1.50~3.24	0.6584	0.95	0.74~1.21	Poor/-		
	Stage	0.0002	1.73	1.29~2.30	0.0028	1.71	1.20~2.44	Poor/Poor		
GSE53963	PLG	0.2355	1.25	0.87~1.79	0.5246	1.05	0.90~1.24	-/-	Yes	Go
	Age	0.1900	1.24	0.90~1.70	0.0596	1.15	0.99~1.33	-/-		
	Stage	0.0003	1.80	1.31~2.48	<0.0001	1.77	1.53~2.04	Poor/Poor		
GSE63885	PLG	0.3064	1.33	0.77~2.28	0.5770	0.91	0.66~1.26	-/-	Yes	Go
	Grade	0.0113	1.71	1.13~2.58	<0.0001	1.66	1.30~2.12	Poor/Poor		
	Stage	0.0084	2.31	1.24~4.32	<0.0001	2.20	1.53~3.17	Poor/Poor		
GSE73614	PLG	0.7245	0.90	0.49~1.64	0.3004	1.15	0.88~1.52	-/-	Yes	Go
	Age	0.0275	1.79	1.07~2.99	0.0003	1.53	1.21~1.94	Poor/Poor		
	Grade	0.0068	1.84	1.18~2.85	0.0007	1.43	1.16~1.76	Poor/Poor		
	Stage	0.0001	1.73	1.30~2.30	<0.0001	1.58	1.39~1.81	Poor/Poor		
GSE8841	PLG	0.7002	0.80	0.25~2.53	0.7603	1.16	0.45~3.00	-/-	Yes	Go
	Age	0.0639	3.02	0.94~9.74	0.7095	1.25	0.38~4.11	-/-		
	Grade	0.0507	2.39	1.00~5.73	0.0232	2.41	1.13~5.17	-/Poor		
	Histotype	0.0364	0.60	0.37~0.97	0.1980	0.79	0.54~1.13	Good/-		
GSE9891	PLG	0.0187	0.52	0.30~0.90	0.5845	0.93	0.71~1.22	Good/-	Yes	Go
	Age	0.1090	1.43	0.92~2.22	0.0190	1.35	1.05~1.74	-/Poor		
	Histological	0.0372	8.14	1.13~58.48	0.0048	5.18	1.65~16.26	Poor/Poor		
	Stage	<0.0001	2.41	1.60~3.61	<0.0001	2.20	1.73~2.80	Poor/Poor		
TCGA	PLG	0.0217	1.41	1.05~1.89	0.0132	1.48	1.09~2.01	Poor/Poor	Yes	Go
	Age	<0.0001	1.56	1.26~1.94	0.0042	1.50	1.14~1.97	Poor/Poor		
	Grade	0.1680	1.23	0.92~1.64	0.3714	1.21	0.80~1.83	-/-		
	Race	0.0986	0.81	0.63~1.04	0.0083	0.66	0.49~0.90	-/Good		
	Stage	0.0008	1.43	1.16~1.76	0.0475	1.36	1.00~1.85	Poor/Poor		

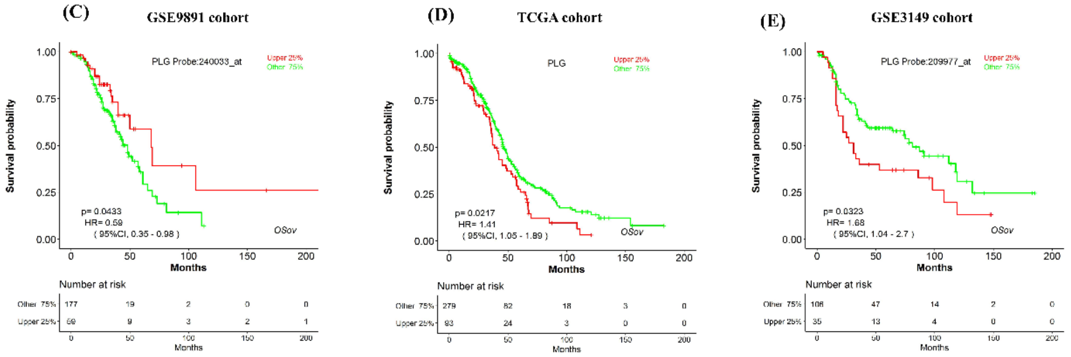

Figure S1. OSov predicts overall survival for *PLG* gene for ovarian patients. Previously reported that overexpression of *PLG* gene is associated with poor survival. (A) the forest analysis for *CRYAB* gene; (B) the univariate and multivariate analysis for *CRYAB* gene; (C) and (D) overexpression of *PLG* gene is a worse predictor in TCGA and GSE3149 cohorts, respectively; (E) a good prognostic biomarker in GSE9891 cohort for ovarian cancer. PLG: plasmin (ogen). Cutoff: upper 25% VS other 75%.
